# Loss of tumour-specific ATM protein expression is an independent prognostic factor in early resected NSCLC

**DOI:** 10.18632/oncotarget.16215

**Published:** 2017-03-15

**Authors:** Lars F. Petersen, Alexander C. Klimowicz, Shannon Otsuka, Anifat A. Elegbede, Stephanie K. Petrillo, Tyler Williamson, Chris T. Williamson, Mie Konno, Susan P. Lees-Miller, Desiree Hao, Don Morris, Anthony M. Magliocco, D. Gwyn Bebb

**Affiliations:** ^1^ Department of Oncology, Tom Baker Cancer Centre and University of Calgary, Calgary, Alberta, T2N 4N2, Canada; ^2^ Functional Tissue Imaging Unit, Translational Research Laboratory, Tom Baker Cancer Centre, Calgary, Alberta, T2N 4N2, Canada; ^3^ Department of Community Health Sciences, TRW Building, University of Calgary, Calgary, Alberta, T2N 4Z6, Canada; ^4^ Gene Function Lab, The Institute for Cancer Research, London, SW3 6JB, UK; ^5^ Department of Biochemistry and Molecular Biology, HRIC Building, University of Calgary, Calgary, Alberta, T2N 4Z6, Canada; ^6^ Department of Anatomic Pathology, H. Lee Moffitt Cancer Center, Tampa, FL, 33612, USA; ^7^ Present Address: Immunology and Inflammation Research, Boehringer Ingelheim Pharmaceuticals, Inc., Ridgefield, CT, 06877, USA

**Keywords:** ATM, non-small cell lung cancer, outcome, early stage, digital pathology

## Abstract

Ataxia-telangiectasia mutated (ATM) is critical in maintaining genomic integrity. In response to DNA double-strand breaks, ATM phosphorylates downstream proteins involved in cell-cycle checkpoint arrest, DNA repair, and apoptosis. Here we investigate the frequency, and influence of ATM deficiency on outcome, in early-resected non-small cell lung cancer (NSCLC). Tissue microarrays, containing 165 formalin-fixed, paraffin-embedded resected NSCLC tumours from patients diagnosed at the Tom Baker Cancer Centre, Calgary, Canada, between 2003 and 2006, were analyzed for ATM expression using quantitative fluorescence immunohistochemistry. Both malignant cell-specific ATM expression and the ratio of ATM expression within malignant tumour cells compared to that in the surrounding tumour stroma, defined as the ATM expression index (ATM-EI), were measured and correlated with clinical outcome. ATM loss was identified in 21.8% of patients, and was unaffected by clinical pathological variables. Patients with low ATM-EI tumours had worse survival outcomes compared to those with high ATM-EI (*p* < 0.01). This effect was pronounced in stage II/III patients, even after adjusting for other clinical co-variates (*p* < 0.001). Additionally, we provide evidence that ATM-deficient patients may derive greater benefit from guideline-recommended adjuvant chemotherapy following surgical resection. Taken together, these results indicate that ATM loss seems to be an early event in NSCLC carcinogenesis and is an independent prognostic factor associated with worse survival in stage II/III patients.

## INTRODUCTION

Recent studies have suggested that non-small cell lung cancer (NSCLC) exhibits a greater degree of genetic instability than most other malignancies [1, 2]. The cause of such genomic instability in NSCLC is not well understood. Although associated with extensive mutagen exposure through smoking in 80% of patients, this genetic instability is surprisingly matched and even exceeded in non-smoking related cases [3]. Genetic heterogeneity, both between and within tumours, has been proposed as a barrier to successful treatment [4] of the advanced disease stage with which most NSCLC patients present, contributing to the disappointing 15% overall five-year survival [5]. While substantial heterogeneity in both the degree and character of such genomic instability has been described [6], it is postulated that dysregulation of the DNA damage response (DDR) is a common means by which such a “mutator phenotype” [7, 8] is generated.

ATM (ataxia-telangiectasia mutated) plays a key role in the cellular DNA damage response (DDR) [9–11], and is emerging as an important tumour-suppressor biomarker in many malignancies, as supported by data suggesting that 15% of gastric cancers carry ATM mutations [12, 13] and that ATM deficiency is prevalent in lymphoid malignancies [14, 15]. Furthermore, in an analysis of listed mutations in 500 human protein kinases in 169 primary tumours and 40 cell lines, mutations in ATM emerged at number 3 in terms of frequency. A large proportion of these mutations were found in lung cancers, which exhibited the highest rate of somatic ATM mutations of all tumours analyzed [16]. Several other studies have confirmed that NSCLC harbors DNA repair deficiencies due to ATM mutations [17]. In two recently published studies, the frequency of ATM deficiency in lung adenocarcinomas was investigated and shown to range from 18% to as high as 40% [18, 19]. The impact on ATM loss was not associated with overall survival [19]. In this particular study the authors assessed total global ATM in patient samples using standard histopathalogical immunohistochemistry (IHC) staining. While clinically valid, this method is not quantitative, nor does it account for tissue heterogeneity within the sample.

To address this, we set out to determine the frequency and clinical significance of loss of ATM expression in a series of early stage, resected cases of NSCLC using quantitative fluorescence IHC. We expect that somatic changes in *ATM* would lead to a loss of ATM expression that could be detected by quantifying the relative expression of ATM protein within the malignant versus the stromal component of the tumour - a concept introduced here as the ATM expression index (ATM-EI). The ATM-EI should be a more stringent measure of ATM loss in tumours, and we hypothesized that increased genomic instability associated with ATM loss [20] in the early stages of NSCLC and would be associated with a worse clinical outcome. Here, we demonstrate that approximately 20% of NSCLC cases show a reduced ATM expression in the malignant compared to the non-malignant components of the tumour and that this loss of ATM expression is associated with a worse prognosis. Furthermore, we present data suggesting that patients with low ATM-EI respond more positively to adjuvant chemotherapy. Together, our data shows that tumour ATM loss has prognostic significance in early stage NSCLC, and could identify a unique cohort of patients who can benefit from disease-modifying therapy.

## RESULTS

### Patient characteristics

To investigate the clinical significance of ATM expression in NSCLC, we measured ATM protein expression in resected tumours. Our investigation adheres to the REMARK criteria for the study of biomarkers [21]. Of 1507 diagnoses of NSCLC between January 2003 and December 2006, 165 patients underwent full resection for stage I, II, or IIIA disease. The majority was classified as stage I (*n* = 104), with 45 stage II and 16 stage III patients. Demographic characteristics and survival were compatible with historical controls. Patient demographics and clinical characteristics are shown in Table 1. Briefly, mean age was 64 years, 53.3% were female, 87.9% were current or ex-smokers, and 6.7% were of Southeast Asian ethnicity; 53.9% were adenocarcinomas, 28.5% squamous cell carcinomas, and 17.6% had other tumour histology (which included bronchoalveolar carcinoma, large cell carcinoma, adenosquamous carcinoma and sarcomatid carcinoma). The majority of the patients (67.2%) underwent a lobectomy as their resection type, and 40.6% (34 stage IB, 11 IIA, 10 IIB, 12 IIIA) received adjuvant treatment. At the end of study date (November 30, 2010), 57.6% of the patients were still alive.

**Table 1:** Demographic characteristics of early stage lung cancer cohort - comparing low and high ATM expression

	Number of Patients^a^ (%)					
	Characteristic	Full Cohort (*n* = 165)	Low Malignant Cell ATM (*n* = 77)	High Malignant Cell ATM (*n* = 88)	Low ATM-EI (*n* = 36)	High ATM-EI (*n* = 129)
Age	Mean (SD)	64.1 (9.8)	64.5 (10.4)	63.7 (9.3)	63.5 (11.1)	64.3 (9.4)
Gender	Male	77 (46.7)	41 (53.3)	36 (40.9)	17 (47.2)	60 (46.5)
	Female	88 (53.3)	36 (46.8)	52 (59.1)	19 (52.8)	69 (53.5)
Ethnicity	South East Asian	11 (6.7)	6 (7.8)	5 (5.7)	2 (5.6)	9 (7.0)
	Other	154 (93.3)	71 (92.2)	83 (94.3)	34 (94.4)	120 (93.0)
Smoking Status	Ever^b^	145 (87.9)	68 (88.3)	77 (87.5)	34 (94.4)	111 (86.1)
	Never	20 (12.1)	9 (11.7)	11 (12.5)	2 (5.6)	18 (14.0)
Histology	Adenocarcinoma	89 (53.9)	43 (55.8)	46 (52.3)	25 (69.4)	64 (49.6)
	Squamous Cell	47 (28.5)	22 (28.6)	25 (28.4)	6 (16.7)	41 (31.8)
	BAC	18 (10.9)	8 (10.4)	10 (11.4)	2 (5.6)	16 (12.4)
	Other^c^	11 (6.7)	4 (5.2)	7 (8.0)	3 (8.3)	8 (6.2)
Stage	I	104 (63.0)	48 (62.3)	56 (63.6)	24 (66.7)	80 (62.0)
	II/III	61 (37.0)	29 (37.7)	32 (36.4)	12 (33.3)	49 (38.0)
Surgical Method	Lobectomy	110 (66.7)	53 (68.8)	57 (64.8)	28 (77.8)	82 (63.6)
	Wedge Resection	35 (21.2)	13 (16.9)	22 (25.0)	5 (13.9)	30 (23.2)
	Pneumonectomy	20 (12.1)	11 (14.3)	9 (10.2)	3 (8.3)	17 (13.2)
Perioperative Chemotherapy	Yes	67 (40.6)	30 (39.0)	37 (42.0)	10 (27.8)	57 (44.2)
	No	98 (59.4)	47 (61.0)	51 (58.0)	26 (72.2)	72 (55.8)

Note: SD = standard deviation.

^a^unless otherwise stated.

^b^includes current, ex, unknown (unknown = 2).

^c^other includes adenosquamous, large cell, sarcomatoid.

Column totals may not add to one hundred due to rounding.

There were no statistically significant differences in characteristics between low and high ATM.

### ATM expression analysis and determination of ATM deficiency

**Figure 1:**
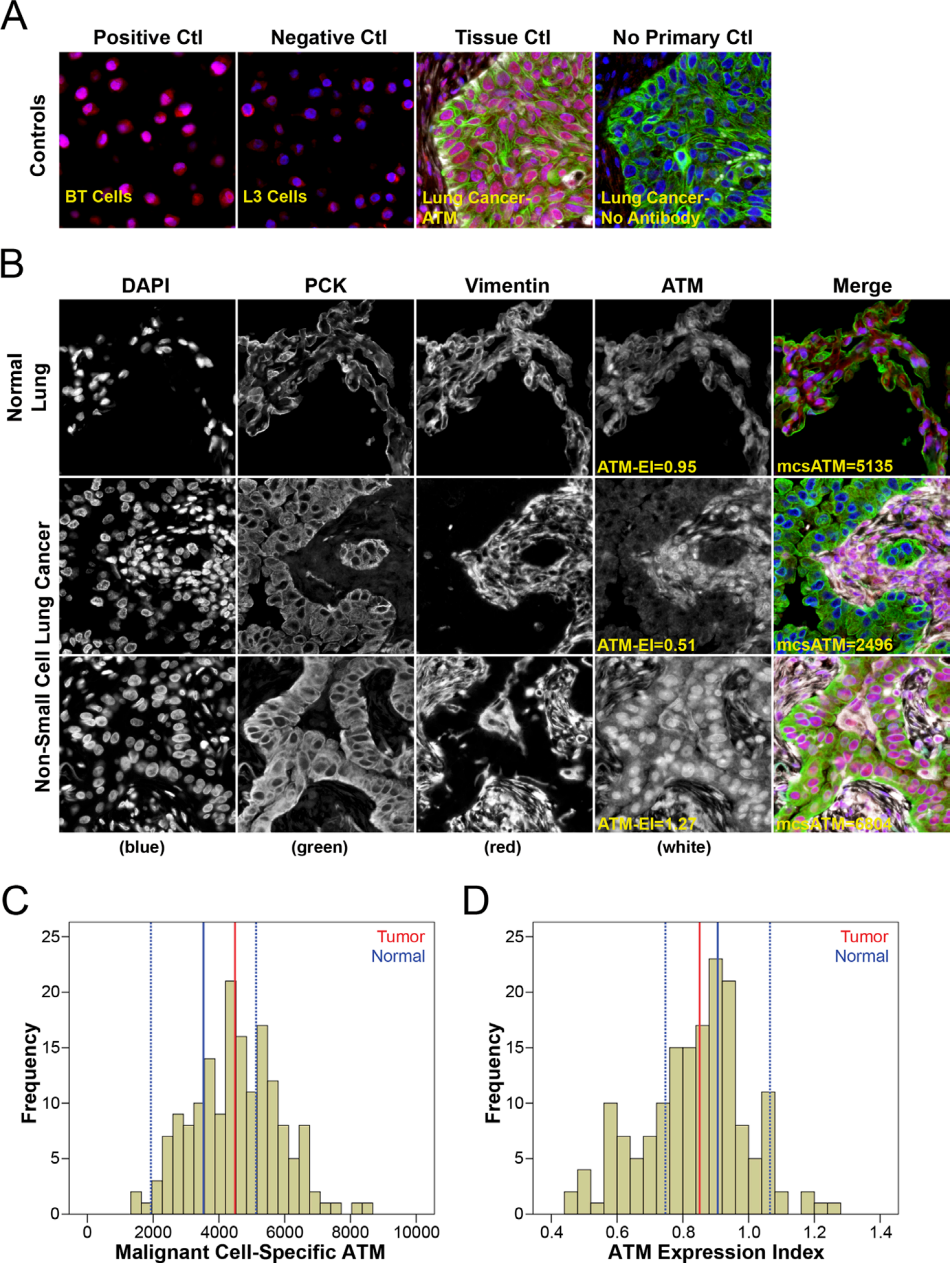
Quantitative fluorescence immunohistochemistry and digital image analysis for ATM in NSCLC (**A**) Control ATM antibody specificity staining in ATM positive BT cells and ATM deficient L3 cells, as well as in lung cancer tissue with and without primary anti-ATM antibody. (**B**) Representative fluorescence images for ATM expression in normal lung tissue and NSCLC. Both ATM expression index (ATM-EI) and malignant cell-specific ATM (mcsATM) scores are reported. DAPI-stained nuclei are depicted in blue, pan-cytokeratin-stained epithelial/malignant cells are depicted in green, vimentin-stained non-malignant tumour stromal cells are depicted in white, and ATM protein expression is depicted in red. Histogram distributions representing malignant cell-specific ATM expression (**C**) and the ATM expression index (**D**) within the NSCLC cohort. The solid blue line represents median ATM expression in normal lung tissue, the broken blue lines represent +/−2 standard deviations from median ATM expression in normal lung tissue, and the solid red line represent median ATM expression in NSCLC.

ATM protein expression levels were measured by quantitative fluorescence immunohistochemistry using the HistoRx AQUA^®^ platform [22] (Figure 1). Antibody specificity was validated using the L3 ATM-deficient human lymphoblastoid cell line and the BT/C3ABR age matched ATM-proficient human lymphoblastoid cell line as controls (Figure 1A, left panels) [23]. ATM expression in normal lung tissue demonstrated a relatively uniform and intense nuclear expression pattern in both epithelial (pan-cytokeratin positive) and non-epithelial cell types (vimentin positive and pan-cytokeratin negative), consistent with the reported cellular distribution of ATM (Figure 1B). In contrast, ATM expression in low-expressing NSCLC samples had only faint and/or sporadic nuclear staining pattern in malignant cells compared with a stronger distinct nuclear staining pattern in adjacent stromal cells (Figure 1B). High ATM-expressing tumours, similar to normal lung tissue, had a strong distinct nuclear expression pattern in both malignant epithelial and stromal cells (Figure 1B).

To determine whether ATM expression was altered in NSCLC compared to normal lung epithelium we compared ATM expression within our NSCLC patient cohort to a 95% confidence interval (C.I.) around the median results from normal lung tissue (Figure 1C and 1D). Malignant cell-specific ATM protein expression was calculated within each patient tissue core as the average ATM pixel intensity within the pan-cytokeratin positive malignant cell area. Median ATM expression in normal lung epithelium was 3531 (95% C.I.: 1931–5130) (Figure 1C, solid and hashed blue lines). Median ATM expression in our NSCLC cohort was 4490 (Figure 1C, red line) and fell in the upper end of the normal lung epithelium 95% C.I., indicating moderate overexpression in tumour tissue.

The ATM-EI was defined as the expression of ATM within the malignant cells of the tumour relative to the ATM expression in the adjacent tumour-associated non-malignant stromal cells. This ATM expression index was calculated by dividing average ATM pixel intensity within the pan-cytokeratin-positive malignant cell area by the average ATM pixel intensity within the corresponding vimentin-positive and pan-cytokeratin-negative tumour-associated stromal area. We hypothesized that the ATM-EI would be a more meaningful measure of ATM deficiency, as it would take into account the heterogeneity of normal ATM expression levels between patients. A low ATM-EI represents a tumour sample in which the tumour portion is ATM deficient relative to the surrounding stromal cells. The median ATM-EI in normal lung epithelium was 0.906 (95% C.I.: 0.747–1.065) (Figure 1D, solid and hashed blue lines). The median ATM expression index in our NSCLC cohort was 0.851 (Figure 1D, red line) and fell in the lower end of the normal lung epithelium ATM-EI 95% C.I., indicating modest under expression in tumour tissue.

### Influence of ATM deficiency on outcome

**Figure 2:**
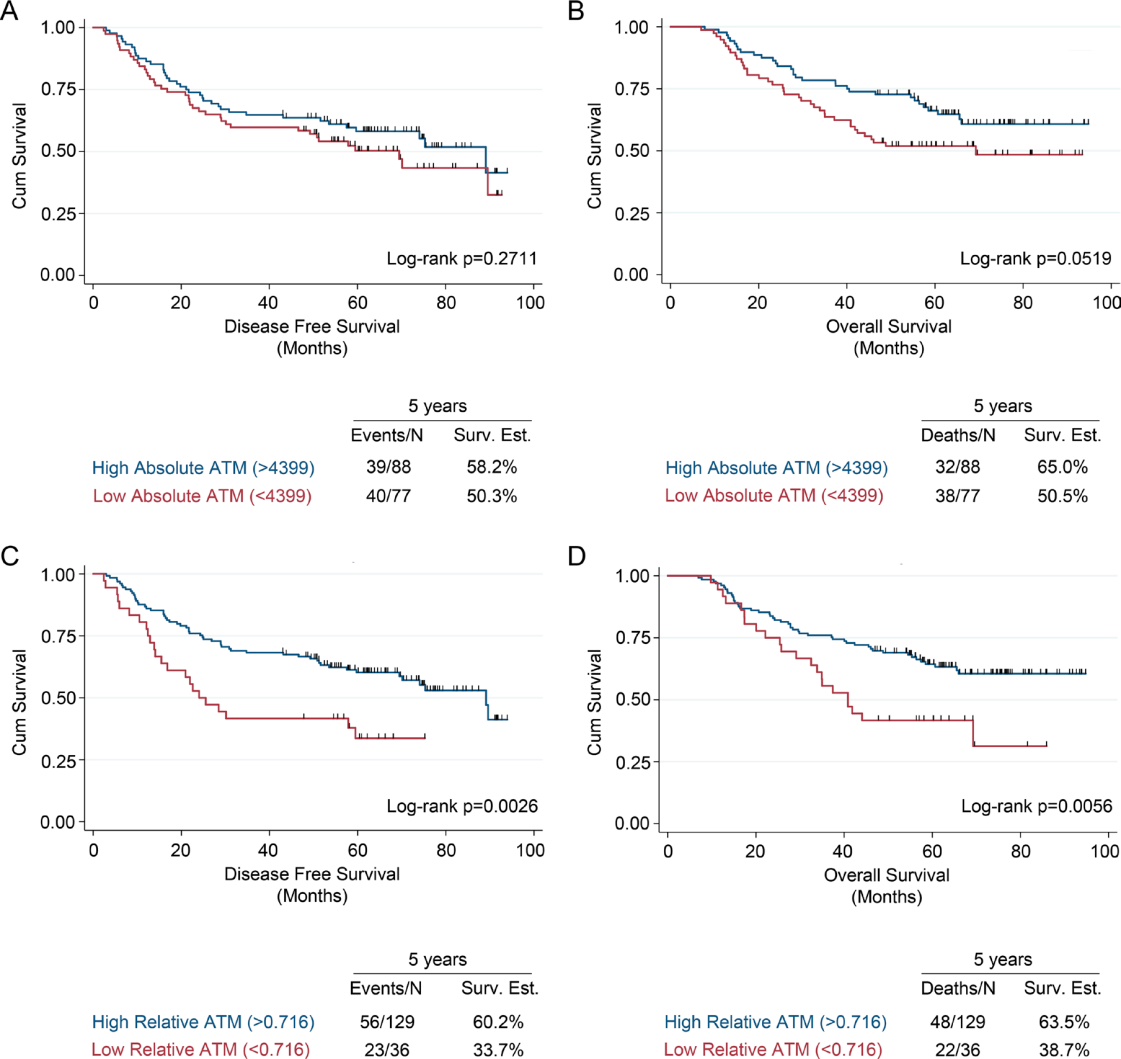
Univariate survival analysis in early stage NSCLC patients based on ATM expression measured using malignant cell-specific ATM and ATM expression index scores Kaplan-Meier analysis and 5-year survival estimates for (**A**) disease free survival and (**B**) overall survival in NSCLC patients with low or high malignant cell specific ATM expression scores. Kaplan-Meier analysis and 5-year survival estimates for (**C**) disease free survival and (**D**) overall survival in NSCLC patients with low or high ATM expression index scores. All Kaplan-Meier curves include the 5-year event rate expressed at the Events/N, where N is the number at risk.

Patients were dichotomized by malignant cell-specific ATM expression and the ATM-EI for survival analysis using the Kaplan-Meier method. Patients were separated into those with a high ATM score and those with a low ATM score using a cut-point based on the log rank statistic [24] (Figure 2). This method identified a malignant cell-specific ATM expression cut-point of 4399, defining 77/165 (46.7%) of patients as being ATM-deficient (Table 1). In contrast, the ATM-EI cut point of 0.716 defined 36/165 (21.8%) of patients as being ATM-deficient (Table 1). There were no significant differences in the clinicopathological features between the high- and low-ATM expressing patients using either method to define the groups (Table 1).

**Figure 3:**
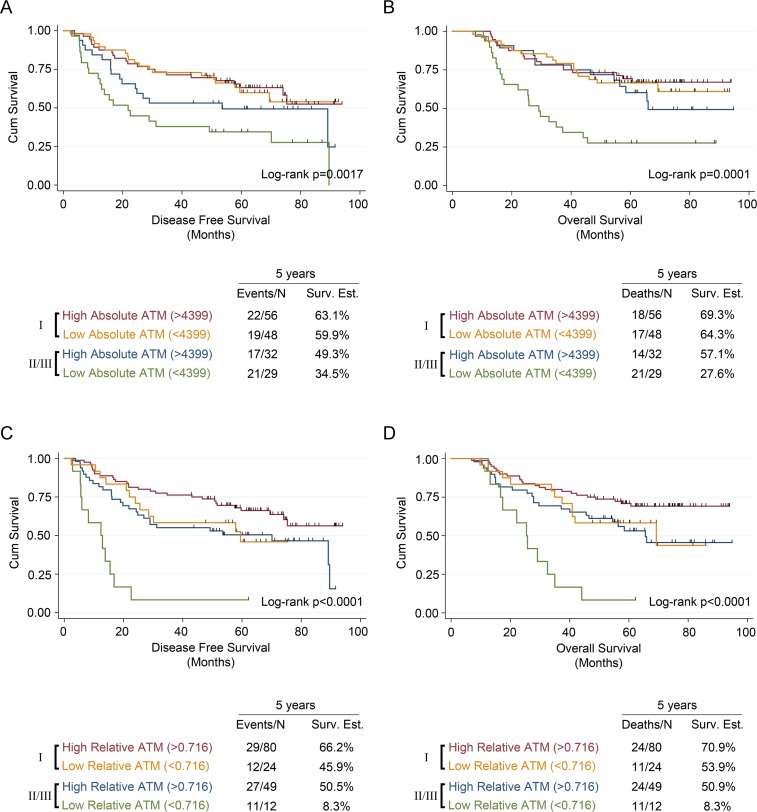
Univariate survival analysis in NSCLC patients stratified by stage and based on ATM expression measured using malignant cell-specific ATM and ATM expression index scores Kaplan-Meier analysis and 5-year survival estimates, stratified into stage I or stages II/III, for (**A**) disease free survival and (**B**) overall survival in NSCLC patients with low or high malignant cell specific ATM expression scores. Kaplan-Meier analysis and 5-year survival estimates, stratified into stage I or stages II/III, for (**C**) disease free survival and (**D**) overall survival in NSCLC patients with low or high ATM expression index scores. All Kaplan-Meier curves include the 5-year event rate expressed at the Events/N, where N is the number at risk.

Compared to patients with a high malignant cell-specific ATM expression, patients with a low ATM expression had worse 5-year disease free survival (DFS) and 5-year overall survival (OS) (Figures 2A and 2B). Similarly, compared with patients that had a high ATM-EI, patients with a low ATM-EI had significantly worse DFS and OS (Figures 2C and 2D). Further analysis showed that the effect of ATM expression levels on DFS and OS is different depending on stage. Among stage I patients, there was no ATM expression effect with either malignant cell-specific ATM or ATM-EI measurements on DFS or OS. However, for stage II/III patients, there is a significant effect on both DFS and OS, using both ATM measurements (Figure 3). As with the overall cohort analysis in Figure 3, the associations between the ATM-EI and survival are stronger than those between malignant cell-specific ATM expression and survival.

Cox proportional hazards models were used to determine if ATM loss identified by either malignant cell-specific ATM expression or the ATM-EI was an independent prognostic factor for DFS or OS. A stage specific approach was adopted for this analysis as stage was shown to modify the ATM survival relationship in the univariate analysis. Neither malignant cell-specific ATM expression nor the ATM-EI demonstrated a prognostic effect for patients with stage I disease (data not shown). However, after adjusting for histology, gender, age, and adjuvant treatment, there was a significant association between the ATM-EI and DFS (HR: 4.75, 95% CI: 2.02 to 11.17, *p* < 0.001) among those with stage II/III disease (Table 2). When examining OS, and after adjusting for the same factors, the ATM-EI was again a significant prognostic factor (HR: 5.09, 95% CI: 2.07 to 12.52, *p* < 0.001) as well as malignant cell-specific ATM (HR: 2.79, 95% CI: 1.38 to 5.66, *p* = 0.004) among those with stage II/III disease (Table 2). Additional analyses explored whether or not the prognostic effect of the ATM-EI was being driven by the stage III patients (*n* = 16). No difference in the ATM effect was observed between the stage II and the stage III patients (DFS - HR = 2.07, 95% CI: 0.32 to 13.48, *p* = 0.45; OS – HR = 1.15, 95% CI: 0.18 to 7.24, *p* = 0.88).

**Table 2:** Cox proportional hazards regression models in early stage lung cancer cohort - comparing low and high ATM expression in stage II/III patients

Factor	5 Year Disease Free Survival		5 Year Overall Survival	
	Hazard Ratio (95% C.I.)	*p*–Value	Hazard Ratio (95% C.I.)	*p*–Value
Malignant Cell-Specific ATM (>4399/<4399)	1.88 (0.96–3.69)	0.068	2.79 (1.38–5.66)	0.004^a^
Adjuvant Treatment (No/Yes)	0.65 (0.33–1.29)	0.222	0.78 (0.39–1.55)	0.476
Adenocarcinoma (No/Yes)	0.40 (0.12–1.31)	0.128	0.59 (0.17–2.02)	0.396
BAC (No/Yes)	0.27 (0.05–1.45)	0.127	0.17 (0.02–1.57)	0.117
Squamous Cell (No/Yes)	0.31 (0.10–0.98)	0.047^a^	0.53 (0.16–1.80)	0.312
Gender (Female/Male)	1.27 (0.62–2.61)	0.514	1.82 (0.87–3.82)	0.113
Age (Continuous)	1.00 (0.96–1.03)	0.873	1.02 (0.98–1.06)	0.250
ATM Expression Index (> 0.716/< 0.716)	4.75 (2.02–11.17)	< 0.001^a^	5.09 (2.07–11.17)	< 0.001^a^
Adjuvant Treatment (No/Yes)	0.69 (0.35–1.34)	0.272	0.87 (0.44–1.71)	0.680
Adenocarcinoma (No/Yes)	0.46 (0.14–1.49)	0.196	0.73 (0.22–2.46)	0.609
BAC (No/Yes)	0.32 (0.06–1.70)	0.182	0.20 (0.02–1.81)	0.151
Squamous Cell (No/Yes)	0.44 (0.14–1.42)	0.171	0.90 (0.26–3.06)	0.863
Gender (Female/Male)	1.21 (0.61–2.42)	0.581	1.81 (0.88–3.74)	0.109
Age (Continuous)	1.01 (0.97–1.05)	0.627	1.04 (1.00–1.08)	0.057

^a^statistically significant to *p* < 0.05.

### ATM deficient patients respond to perioperative chemotherapy

**Figure 4:**
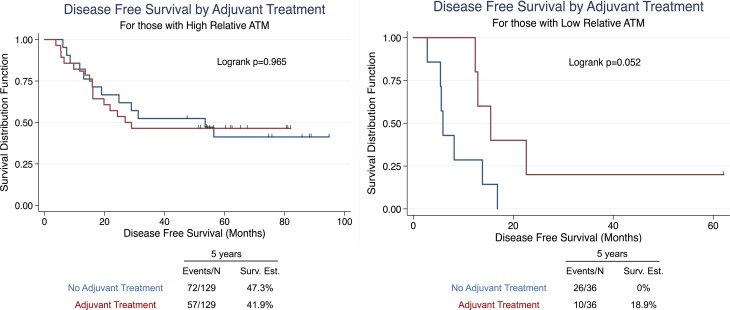
Univariate survival analysis in NSCLC patients stratified by adjuvant treatment in high ATM-EI and low ATM-EI groups Kaplan-Meier analysis and 5-year disease free survival estimates, stratified into Adjuvant Treatment or No Adjuvant Treatment for (**A**) high ATM expression index, and (**B**) low ATM expression index scores. All Kaplan-Meier curves include the 5-year event rate expressed at the Events/N, where N is the number at risk.

To assess the therapeutic impact of low ATM EI in this setting we compared outcomes among those patients who received perioperative systemic therapy. Periopertative chemotherapy (platin/vinorelbine combination chemotherapy) was given to 40.6% (67/165) of patients in our cohort of whom 64 received adjuvant chemo, 1 received neoadjuvant chemo, and 1 received neoadjuvant chemo/radiation therapy. When sorted into high and low ATM-EI groups, patients with low ATM-EI treated with platin based perioperative treatment showed a strong trend toward improved disease free survival, (10/36; *p* = 0.052), while there was virtually no difference in DFS among treated or untreated patients with high ATM-EI (57/129; *p* = 0.965). This suggests that low ATM EI may be predictive of benefit from adjuvant platin based treatment. (Figure 4).

## DISCUSSION

The key role held by ATM in maintaining genomic integrity as part of the DDR suggests that early loss of ATM function in the process of malignant transformation could herald the generation of a mutator phenotype, an “enabling feature” in the evolution of cancer [25]. Our findings on clinical outcome in patients diagnosed with early resectable NSCLC lend support to the hypothesis that ATM loss is an important event in NSCLC carcinogenesis.

By using the digital image analysis capability of the HistoRx AQUA^®^ platform, we were able to quantify ATM protein levels and to define the specific localization of ATM expression within the tumour. Using the ratio of ATM expression in the malignant component compared to the stromal elements of the tumor (the ATM-EI), we defined a cohort of 22% of patients whose NSCLC tumors could be described as being ATM-deficient. Interestingly, there was no difference in the incidence of relative ATM deficiency between stage I and stage II/III patients. This methodology for determining ATM-deficiency proved more stringent than standard histopathology staining, defining 22% of patients in our cohort as ATM-deficient, compared to 40% deficiency in a similar-sized cohort, recently reported by Villaruz *et al*. [19]. Similarly, quantitative IHC of ATM in breast cancer identified a smaller subgroup of ATM-deficient patients [26] than does standard histopathology [27], however in both of these studies ATM-loss was associated with poor DFS.

By using the accompanying outcome data in the Glans-Look lung cancer database, we confirm that measuring absolute ATM level in samples does not significantly predict survival in early-resected NSCLC, as was previously reported [19], however we show that when ATM deficiency is defined as a ratio of tumour to stromal expression below 0.716, ATM loss is an independent predictor of both 5-year DFS and 5-year OS. This analysis is arguably more biologically relevant, as the genomic instability induced by ATM loss specifically in the cancerous cells could drive progression of the tumor. Certainly, variable expression of biomarkers including ATM in cancer-associated stromal tissues have been observed [26, 28], and higher levels are associated with aggressive characteristics of breast cancer [26]. While stromal ATM expression in our samples did not predict outcome (not shown), the ATM-EI value does consider the stromal compartment in the calculation.

The fact that relative ATM deficiency is seen in a proportion of stage I patients suggests that reduced ATM expression is an early event in NSCLC carcinogenesis and supports the hypothesis [29] that tumour progression is associated with a disruption in the ATM-CHK2-p53 pathway in many human cancers, including lung cancer. Intriguingly, there was no clear association between ATM deficiency and smoking, perhaps indicating that disruption in the ATM-CHK2-p53 pathway is one of several mechanisms by which lung carcinogenesis can occur. An association between genomic instability and increased immunogenicity has been proposed. Recent data suggest that immune-modulating treatments have a role to play in NSCLC where efficacy is seen regardless of smoking status [30, 31]. Our data therefore lends a mechanistic basis for this observation.

Perhaps more important from a patient perspective is our observation that adjuvant treatment positively influences the 5-year DFS of the low ATM-EI group, suggesting that ATM-deficiency may increase sensitivity to certain chemotherapies. This is consistent with recent reports and our own data (not shown) that have shown increased platin-induced cytotoxicity *in vitro* in ATM- and DDR-deficient cell lines [32, 33]. Additionally, ATM-loss in breast cancer If this is the case, our data suggests that while patients with early stage ATM-proficient NSCLC receive little-to-no advantage from adjuvant therapy, those with ATM-deficient tumours could benefit greatly under current standard treatment regimens, or even low dose therapy.

Our study has inherent limitations. It did not set out to investigate the mechanisms underlying the loss of ATM expression in NSCLC. In addition to the many *ATM* mutations described in the literature, several other mechanisms for altering or silencing ATM have been delineated [34]. Furthermore, a simple measure of ATM protein expression by IHC cannot establish the functional capability of ATM within the tumour. It is possible that in some tumours that are shown to be ATM deficient by IHC, ATM functionality is still preserved, while in other cases ample ATM expression by IHC could be detecting non-functional, truncated protein. Other studies suggest that p53 loss may further influence the role of ATM deficiency on cellular phenotype including chemosensitivity [23]. Clearly, this represents a therapeutically exploitable deficit and may help target the use of non-specific DNA damaging agents such as ionizing radiation, cytotoxic drugs and more targeted agents such as PARP inhibitors in future treatment algorithms for advanced NSCLC.

In summary, we report that loss of ATM expression, as denoted by a low ATM-EI within the malignant component of a tumour, can be detected in a substantial proportion of resected NSCLCs. Such loss of ATM is seen in many stage I tumours, implying that this is a relatively early event in NSCLC tumourigenesis. Our analysis also suggests that a low ATM-EI confers a poor prognosis in terms of both DFS and OS on patients despite resection. The implications of ATM loss may also have a predictive utility since several therapeutic modalities used in NSCLC may exploit this deficiency. Investigations that determine the underlying mechanisms of ATM loss may reveal different molecular pathways of carcinogenesis that ultimately lead to NSCLC. Meanwhile, further clinical studies on the association between this therapeutically important protein and outcome and response, in early and advanced stage NSCLC, will be important to improve the targeting and efficacy of existing treatment modalities.

## MATERIALS AND METHODS

### Case selection and clinical data collection

This study was approved by the University of Calgary Conjoint Faculties Research Ethics Board, in accordance with the Tri-Council Policy Statement on Research with Human Subjects. Clinical data was collected retrospectively through chart review of NSCLC patients diagnosed at the Tom Baker Cancer Centre from 2003 to 2006 and entered into the Glans-Look Lung Cancer Database as previously described [35]. Tumour resection type was divided into wedge resection, lobectomy and more extensive surgery (bi-lobectomy, hemi-pneumonectomy, or a pneumonectomy). Adjuvant treatment included patients who received adjuvant chemotherapy alone, adjuvant chemoradiotherapy, and those who received neoadjuvant radiotherapy.

### Tissue microarray generation

All archived formalin-fixed paraffin-embedded (FFPE) resected NSCLC tumour samples were retrieved from Calgary Laboratory Services and reviewed by a pathologist (AMM). Tissue microarrays where constructed as previously described [35]. Normal lung tissue specimens (*n* = 21), normal tonsil tissue, an ATM-deficient human lymphoblastoid cell line (L3) and an age matched ATM-proficient human lymphoblastoid cell line (BT/C3ABR) were also included as controls [23].

### Four-colour fluorescence immunohistochemistry

Sections (4 μm-thick) from each TMA block were processed and stained as previously described [35, 36]. Slides were processed using a Dako Autostainer and incubated for 60 minutes with a cocktail of three primary antibodies, including: anti-pan-cytokeratin to identify tumour cells, anti-vimentin to identify stromal cells, and anti-ATM (described in Supplementary Table 1). The anti-ATM antibody was previously validated and characterized [19]. Protein expression was visualized with consecutive applications of secondary antibodies and fluors as previously described [35].

### Automated image acquisition and analysis

Automated image acquisition was performed using a HistoRx PM-2000™, previously been described in detail [22]. Briefly, high resolution monochromatic 8-bit digital images were obtained for every histospot on the TMAs using filters specific for DAPI, FITC to define cytokeratin positive NSCLC cells, Cy3 to define vimentin positive non-malignant stromal cells, and Cy5 to define the target biomarker ATM. Images were then analyzed using the AQUAnalysis^®^ program, version 2.2.1.7 as previously described [36].

### Assessment of ATM deficiency

The average intensity of target ATM signal in the masked areas was tabulated and used to generate malignant cell-specific, and non-malignant tumour-associated stromal cell-specific AQUA scores, which reflect the average signal intensity per malignant area, and stromal area, respectively. The ATM expression score was defined as the minimum ATM malignant cell-specific AQUA score from the triplicate cores for each patient sample. The ATM expression index was defined as the minimum ratio, from the triplicate cores for each patient sample, of the malignant cell-specific AQUA score as compared with the non-malignant tumour-associated stromal cell-specific AQUA score.

### Statistical analysis

Cut-points to identify two groups from the minimum ATM expression score and the minimum ATM expression index levels were found using a method based on the log-rank test statistic [24]. Differences in demographic and clinicopathological characteristics were assessed across the two groups using chi square tests for categorical data and *t*-tests for continuous data. Differences in the survival were compared using the log-rank test for low versus high ATM expression index groups and ATM expression groups as well as the stage subgroups. Cox proportional hazards regression was used to assess the prognostic effect of both ATM expression index and tumour ATM. Demographic and clinicopathological factors were included in the multivariable Cox proportional hazards models using an epidemiological framework. Variables were first assessed as potential modifiers and in the absence of the modification variables were assessed for their potential effect as confounders of the investigation under consideration. Proportional hazards assumptions were examined and confirmed by log-negative log Kaplan-Meier curves and by inspecting interaction terms of log (survival time) by the covariates of interest. Five-year disease free and overall survival estimates were calculating using the Kaplan-Meier method. All statistical analyses were conducted using SAS/STAT software (Version 9.2, SAS Institute Inc, Cary, NC, USA).

## SUPPLEMENTARY MATERIALS TABLES


