# Supplementary material for: Loss of tumour-specific ATM protein expression is an independent prognostic factor in early resected NSCLC
Source: Oncotarget. 2017 Mar 15;8(24):38326–36. doi: 10.18632/oncotarget.16215 (PMC5503535; doi:10.18632/oncotarget.16215)
Supplement: Supplementary file 1 [file oncotarget-08-38326-s001.pdf]

Loss of tumour-specific ATM protein expression is an independent prognostic factor in early resected NSCLC

Supplementary Materials

Supplementary Table 1: Antibodies for immunohistochemical (IHC) analysis

Antigen	Species	Primary dilution	Company
ATM	Rabbit monoclonal	1:200 (IHC)	Epitomics
Pan-cytokeratin	Guinea pig polyclonal	1:100 (IHC)	Acris
Vimentin	Rat monoclonal	1:500 (IHC)	R&D
